# Cognitive Training for Emotion-Related Impulsivity and Rumination: Protocol for a Pilot Randomized Waitlist-Controlled Trial

**DOI:** 10.2196/54221

**Published:** 2025-02-19

**Authors:** K J D Allen, Matthew V Elliott, Eivind Haga Ronold, Liam Mason, Nandini Rajgopal, Åsa Hammar, Sheri L Johnson

**Affiliations:** 1 Department of Psychology University of California, Berkeley Berkeley, CA United States; 2 Department of Biological and Medical Psychology University of Bergen Bergen Norway; 3 Department of Clinical, Health & Educational Psychology Division of Psychology and Language Sciences University College London London United Kingdom; 4 Department of Clinical Sciences Lund Psychiatry, Faculty of Medicine Lund University Lund Sweden; 5 Office for Psychiatry and Habilitation Psychiatry Research Skåne Skåne Sweden

**Keywords:** cognitive control, cognitive training, emotion regulation, emotional response inhibition, emotional working memory, executive function, impulsivity, rumination, transdiagnostic, urgency

## Abstract

**Background:**

Inhibitory deficits are common in psychopathology. Emotion-related impulsivity (ERI) and rumination are general risk factors for psychiatric distress that are similarly associated with dysfunctional inhibition—particularly in affective contexts. A number of cognitive remediation procedures have been developed to improve inhibitory control; however, most remediation programs focus on “cold” cognition independent of affective processing. This pilot trial will gather preliminary evidence for a new cognitive training intervention targeting “hot” affective control (ie, inhibitory functions during elevated emotional arousal) in a transdiagnostic sample of adults who report heightened emotion dysregulation.

**Objective:**

This manuscript describes a protocol for a pilot randomized waitlist-controlled trial to assess changes in ERI and rumination after neurobehavioral affective control training (N-ACT), an 8-week cognitive training intervention designed to improve emotional response inhibition and emotional working memory. Our primary aim is to evaluate the efficacy, feasibility, and acceptability of N-ACT in reducing rumination and ERI, which we respectively conceptualize as complementary cognitive and behavioral consequences of emotion dysregulation. Secondarily, we will examine whether N-ACT leads to improvements in inhibitory control and, more distally, psychopathology symptoms.

**Methods:**

The final sample will comprise 80 adults who report high ERI or rumination. Participants will be randomized to (1) begin the N-ACT program without delay or (2) join a waitlist condition and then complete N-ACT. Exclusion criteria include active alcohol or substance use disorders, psychosis, and suicide risk. At the baseline and postintervention time points, participants will complete measures of emotion dysregulation and psychiatric symptoms, as well as a neuropsychological assessment of inhibitory control. Individuals assigned to the control group will undergo an identical assessment before joining the waitlist, followed by parallel assessments before and after N-ACT.

**Results:**

This trial is funded by support from the University of California Board of Regents and the Peder Sather Foundation (funding period: October 2022-September 2025). Recruitment is scheduled to begin in spring 2025. We will begin data analysis once data collection is complete, which is planned to occur in fall 2025.

**Conclusions:**

This pilot randomized waitlist-controlled trial is designed to assess the initial efficacy, feasibility, and acceptability of N-ACT, a novel cognitive remediation approach developed to address 2 key contributors to psychopathology: ERI and rumination. The N-ACT program uses computerized adaptive behavioral tasks to strengthen the affective control processes theoretically and empirically linked to ERI and rumination. We hope this work will help inform future studies with sufficient statistical power to ascertain whether enhancing affective control through cognitive training (N-ACT) produces downstream reductions in psychiatric symptoms via improved emotion regulation.

**Trial Registration:**

ClinicalTrials.gov NCT06226467; https://www.clinicaltrials.gov/study/NCT06226467; Open Science Framework Registry rak5z; https://osf.io/rak5z

**International Registered Report Identifier (IRRID):**

PRR1-10.2196/54221

## Introduction

### Background

Emotion dysregulation is an established risk factor for nearly all psychiatric syndromes, with particularly strong effects for anxiety and depression—the 2 most common psychological disorders [1,2].These links between emotion dysregulation and psychiatric dysfunction are well documented across ecological and laboratory studies, as well as in large-scale prospective work [3-5]. This research will evaluate a novel intervention, neurobehavioral affective control training (N-ACT), which is designed to target two trait-like facets of emotion dysregulation: (1) *rumination*, the tendency toward self-focused, past-oriented, repetitive negative thinking about the causes and consequences of negative affect [6,7]; and (2) *emotion-related impulsivity* (ERI; [8]), the tendency toward reduced behavioral control during states of high affective arousal [9], most commonly captured using measures of Urgency. These 2 aspects of emotional dysregulation reflect cognitive and behavioral responses, respectively, to negative affect. Robust associations have been documented of rumination and ERI with anxiety and depressive disorders as well as numerous other psychiatric conditions [6,7,10-18].

### Inhibitory Control Deficits in Psychopathology

Rumination and ERI are both tied to problems with cognitive (inhibitory) control. Cognitive control is a multifaceted construct that includes (1) the ability to update and manipulate the contents of working memory; and (2) the inhibition of task-inappropriate behavior (ie, response inhibition), alongside closely related executive functions (eg, cognitive inhibition, set shifting or task switching). This project targets both working memory and response inhibition, which represent promising candidate mechanisms linking emotion regulation deficits to varied manifestations of psychopathology.

Cognitive difficulties associated with major depressive disorder (MDD) and other internalizing syndromes often have severe consequences for the individuals affected, likely through adverse influences on symptoms and role functioning. Systematic reviews of this literature suggest that cognitive control deficits in MDD are not simply epiphenomenal effects caused by symptoms—as some have recently suggested [19]—but rather often persist after remission, thereby exerting continued influence on everyday functioning and the likelihood of symptom recurrence [20,21]. Indeed, impaired cognitive control predicts relapse in patients with first-episode depression [22] up to 5 years after the index episode through its association with rumination [23,24]. One of the longest prospective studies of cognition in MDD revealed cognitive control impairment a full decade after symptom remission [25]. In sum, a substantial literature implicates poor cognitive control in prolonged vulnerability to depression recurrence and relapse.

Evidence for chronic neuropsychological deficits in MDD and other psychiatric disorders has generated interest in improving mental health through strengthening cognitive control and other executive functions. However, traditional psychotherapeutic interventions have limited effects on cognitive control; for example, meta-analyses indicate little to modest changes in cognitive control capacities after psychological [26] or pharmacological treatment for depression [27]. The recognition of the need for new approaches to enhancing executive functions has led to a growing focus on development of cognitive remediation or training programs that directly target inhibitory control [28-30].

However, many studies—of major depression or otherwise—rely on measures of “cold” cognitive control, in that the assessment procedures lack emotionally salient stimuli or probes. *Affective control* refers to the ability to exert inhibitory control in situations or tasks involving emotionally arousing content, often referred to as “hot” executive functions. Emotional contexts may be more likely to activate or aggravate symptoms compared to contexts without such affective components [31]. Rumination and ERI—as well as psychopathology more broadly—may be more closely linked to affective control deficits than to cold cognitive control deficits. For example, among people with remitted MDD compared to healthy controls, Ronold et al [32] observed working memory deficits (during high cognitive load) especially for negative compared to positive emotional content; moreover, in 2 independent samples, such impairment was tied to heightened rumination [33], which in turn predicted elevated risk for depressive relapse [32]. ERI is similarly associated with impaired inhibitory control, particularly during higher arousal states [34]. Moreover, poor affective control is associated with symptoms of eating disorders [35], nonsuicidal self-injury [36-40], and suicidal behaviors [31,41,42]. Thus, affective control is an empirically justified and theoretically informed target to remediate and prevent the recurrence of psychiatric symptoms.

A growing corpus of research has converged on 2 components of affective control, both of which are targeted by N-ACT: (1) *emotional working memory*, which involves the temporary storage and manipulation of emotional information in short-term memory; and (2) *emotional response inhibition*, or the ability to regulate motor impulses driven by automatic emotional reactions. Dysfunction in working memory or response inhibition—*specifically in the context of negative emotion*—is independently tied to rumination, ERI, and internalizing symptoms, as well as self-injurious thoughts and behaviors [39,41,43-46]. Several studies have shown that emotional response inhibition, as indexed by an emotional stop-signal task (ESST), shows significant correlations with measures of ERI and psychiatric severity [37,39,43]. Negative emotional response inhibition impairment is a strong predictor of symptoms, including increased risk of future suicide attempts [41].

### Cognitive Remediation in Psychiatry

The goal of cognitive remediation is to improve dysfunctional neurocognitive processes. This improvement relies on the repeated practice of specialized cognitive training exercises using computer algorithms that adjust task difficulty according to user performance. A burgeoning literature suggests that cognitive remediation may be a promising transdiagnostic intervention approach [28,47-55]. Working memory training has been found to reduce the recurrence of mood episodes among patients with remitted MDD, with stronger protective effects—apparent up to 2 years after the intervention—for those who reported reduced rumination after training [52,56]. Although accumulating evidence supports the efficacy of cognitive remediation in psychiatric disorders, meta-analytic effect sizes tend to be small to medium [28], and a substantial proportion of individuals do not respond to existing training programs for symptom reduction [57].

Numerous issues likely contribute to the relatively modest treatment effects associated with standard cognitive remediation programs. N-ACT is novel in 3 ways. First, we focus on emotion regulation rather than psychiatric symptoms as our primary outcome. Second, extant literature shows considerable variability in the delivery methods of cognitive remediation. Following consensus recommendations from an expert working group [47], we will implement scaffolding techniques to facilitate the generalization and functional impacts of N-ACT. More specifically, we will follow recommendations to provide support to facilitate translation of skills into everyday activities, so as to enhance the ecological validity and effectiveness of cognitive training programs [47-55]. Participants will accordingly complete N-ACT intervention sessions guided by a “coach,” who will deliver semistructured and (partially) personalized psychoeducational content. N-ACT coaching sessions will include the identification of compensatory problem-solving strategies for emotion regulation, skills practice, motivational enhancement, discussion of the ecological relevance of the cognitive skills being trained, and procedures to promote the transfer of training effects to real-world situations. Therefore, we will be testing the effects of repeated affective control exercises, coupled with therapeutic coaching. Our hope is that this combination of techniques will support generalization to improved functional outcomes.

Third, although the stimuli and parameters in many cognitive control training tasks (eg, those using geometric shapes and letters) lack inherent salience value, our trial builds from a smaller body of work on cognitive remediation using the affective variants of inhibitory control tasks as well as traditional tasks (with neutral stimuli) that may naturally induce emotional arousal [50]. As an example of the latter, the Paced Auditory Serial Addition Test [58], which uses numerical stimuli and has been extensively implemented as an adaptive training task [29,30,59], characteristically evokes frustration [58]. To our knowledge, the only direct comparison of cold versus hot cognitive remediation was performed in a pilot trial of emotional working memory training, which yielded promising results in symptoms and cognition (but not rumination) among patients with depressive symptoms [60]. This early work is consistent with recent meta-analytic findings suggesting that hot inhibitory control training may be more effective than nonaffective cognitive remediation procedures for psychopathology [61]. Accordingly, N-ACT incorporates affective stimuli (eg, naturalistic images with standardized emotional content) into traditional training tasks designed to improve cognition such that users learn to use these mental operations in socioemotional contexts that more closely match in vivo experiences. The inclusion of affective stimuli is particularly important, given a substantial body of work suggesting that difficulties sustaining inhibitory control during states of elevated arousal are particularly relevant to emotion regulation and psychiatric symptoms.

### This Study

#### Overview

This pilot randomized waitlist-controlled trial will enroll adult participants with high rumination or ERI scores. The primary aim of this study is to examine the acceptability, feasibility, and efficacy of N-ACT as a novel therapeutic approach to reduce rumination and ERI by improving 2 facets of affective control: emotional working memory and emotional response inhibition. Secondarily, we will test the effects of N-ACT on behavioral indices of ability transfer as well as on other subjective measures of emotion dysregulation (beyond trait rumination and ERI) and psychopathology symptom severity (refer to Multimedia Appendix 1 for details). Finally, we will conduct exploratory analyses to examine the potential mechanisms that might influence predicted changes in emotion dysregulation, informing future work to systematically test these effects in a sufficiently powered sample. In addition to performing intent-to-treat analyses, we will conduct sensitivity analyses to evaluate the extent to which program adherence predicts the hypothesized effects.

#### Hypotheses

We propose the following hypotheses:

- H1: Participants will rate N-ACT as an acceptable intervention to reduce emotion dysregulation.
- H2: Participants will demonstrate adherence to the N-ACT program at levels comparable to those observed in established cognitive remediation protocols [33,52,62-64].
- H3 (primary): Compared to baseline, participants assigned to complete N-ACT (without a waitlist) will report greater decreases in rumination and ERI at the postintervention assessment than those assigned to the control condition at the postwaitlist assessment.
- H4 (secondary): Compared to baseline, participants assigned to complete N-ACT (without a waitlist) will demonstrate greater improvements in targeted affective control processes (ie, enhanced emotional working memory and emotional response inhibition) at the postintervention assessment than those assigned to the control condition at the postwaitlist assessment.
- H5 (secondary): Compared to baseline, participants assigned to complete N-ACT (without a waitlist) will demonstrate near (ie, enhanced hot affective flexibility) and far (ie, enhanced cold working memory and cold response inhibition) transfer of trained neurocognitive abilities at the postintervention assessment than those assigned to the control condition at the postwaitlist assessment.
- H6 (secondary): Compared to baseline, participants assigned to complete N-ACT (without a waitlist) will report greater decreases in self-rated psychopathology symptom severity (ie, reduced internalizing and externalizing symptoms) at the postintervention assessment than those assigned to the control condition at the postwaitlist assessment.

## Methods

### Overview

This research comprises a delayed intervention randomized waitlist-controlled trial with a mixed, 2-group (2 between-subject levels: active vs control), repeated measures (2 within-subject levels: T1=baseline or preintervention time point; T2=postwaitlist or postintervention time point) design, for which we will recruit English-speaking adults (aged 18-65 y) from the San Francisco Bay area of the United States. The inclusion criteria include high self-reported rumination or ERI. Eligible individuals will be invited to the university for an initial baseline session. At this session, they will complete informed consent procedures and an assessment battery consisting of computerized neuropsychological tasks with concurrent psychophysiological monitoring. After the baseline session, participants will complete a battery of questionnaires, followed by 1 week of baseline ecological momentary assessment (EMA) of real-world daily fluctuations in mood, rumination, and ERI.

**Figure 1:**
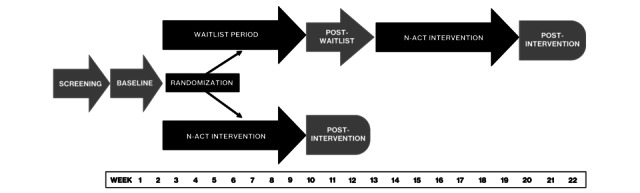
Flow of participants through the trial with a delayed intervention randomized waitlist-controlled design. N-ACT: neurobehavioral affective control training. Assessments are indicated in gray.

After the baseline assessment (laboratory session, questionnaires, and EMA), we will randomly assign participants to receive active treatment (ie, N-ACT without delay) or the waitlist control condition, unblinded, using an asymptotic maximal procedure to achieve a maximally tolerated imbalance of 2, while maintaining a 1:1 allocation ratio between the 2 trial arms [63]. Participants in the active N-ACT group will begin the intervention, which consists of 8 weekly coached training sessions, within 1 week after baseline EMA. We will recontact waitlist control participants after a period equivalent to the intervention duration (ie, 2 months) to complete a second (postwaitlist) assessment session and EMA before starting the N-ACT program for the following 8 weeks. After the intervention, participants will be asked to complete a second week of EMA, and then a postintervention assessment session with comparable measures to baseline. We will recontact waitlist control participants approximately 10 weeks after their (prewaitlist) baseline assessment session to complete a second (postwaitlist) assessment and 1 week of EMA, parallel with the pre-intervention assessment procedures of the N-ACT group,. The waitlist group will then be offered 8 weeks of N-ACT, followed by a second week of postintervention EMA, and then a final assessment session. In sum, we will follow participants assigned to receive the intervention without delay for 12 weeks (3 mo) and those assigned to the waitlist control group for 22 weeks (5.5 mo). This delayed intervention randomized waitlist-controlled trial design enables us to offer all interested participants the opportunity to participate in the program and maximizes our ability to rapidly accrue a sufficiently sized sample for analyses. Figure 1 demonstrates the flow of potential participants through the study procedures.

Hypotheses and analyses were preregistered at OSF. We plan to detail any changes to this protocol in future publications and disseminate our findings to the scientific community in accordance with the CONSORT-EHEALTH (Consolidated Standards of Reporting Trials of Electronic and Mobile Health Applications and Online Telehealth) checklist (V 1.6.1) [64].

### Ethical Considerations

The University of California Berkeley Committee for Protection of Human Subjects granted approval for this research (2023-01-15949) before data collection. All participants will complete written informed consent before commencing study procedures.

Participants will be assigned an ID number, which will be used in place of their name or other identifying information on all research materials. Data will be kept in secure, password-protected files. Although we plan to make data publicly available for reanalysis, we will carefully deidentify data before doing so. In publishing or presenting the results, we will not provide any information that could identify an individual participant.

Participants will receive course credit or monetary compensation for completing assessment procedures but will not be paid for time spent on screening or the intervention itself. Students will be able to earn credit toward required research hours in their psychology classes. We plan to increase the hourly payment rate slightly over the course of the trial to promote retention, from US $20/h for the baseline assessment to US $30/h for the postwaitlist and postintervention assessments. We will compensate participants up to US $60 (or 3 h of course credit) for completing the baseline assessment (2.5-h in-person session plus 30 min of questionnaires), a US $10 “bonus” for performance on an unrelated computer task during the baseline session, US $10 for each week of EMA if they complete at least 75% of the prompts, and US $60 for the postintervention assessment (1.5-h in-person session plus 30 min of questionnaires). Those randomized to the waitlist control condition will be eligible to receive up to an additional US $60 for completing the postwaitlist assessment (identical to the postintervention assessment).

In sum, participants assigned to complete N-ACT without the waitlist can earn up to US $150 (or 3 h of course credit plus up to US $90), while those in the waitlist control condition could earn up to US $70 in addition to those amounts. Payments will be prorated if participants complete only part of an assessment. We will remunerate participants within 1 week of each completed assessment via digital credit cards, which will be replenished as necessary.

### Study Procedures

#### Recruitment and Screening

We will invite up to 500 adults to complete preliminary screening procedures and an estimated 100 participants to complete the initial baseline assessment session. After accounting for 20% expected attrition, we expect 40 participants in each condition to complete all study procedures.

Study advertisements will be posted locally in public spaces and distributed by web (eg, via email listserves, Craigslist, and social media sites). We will also administer a prescreening survey to students enrolled in psychology classes at the University of California Berkeley, and we will invite students who meet the preliminary inclusion criteria based on those responses to take part in the study. Potential participants (recruited either from the community or from the student participant pool) will be directed to our website, where they can view additional details about the trial. We intend to recruit only secondarily from the student participant pool to supplement community-based recruitment, which will constitute most of the targeted sample. All questionnaires, including screening items, will be administered through REDCap (Research Electronic Data Capture; Vanderbilt University) software [65], which will also be used for the secure storage of collected data.

After providing informed consent, interested individuals will complete the brief web-based screening via REDCap to assess preliminary eligibility. The inclusion and exclusion criteria are presented in Textbox 1.

**Inclusion and exclusion criteria.**

**Inclusion criteria**

- Current California residency
- Age 18 to 65 y
- Elevated scores on self-report measures of rumination (Mn >2 on the Brooding subscale of the Ruminative Responses Scale [13]) or emotion-related impulsivity (Mn >3 on the Feelings Trigger Action factor of the Three-Factor Impulsivity Index [9])

**Exclusion criteria**

- Insufficient English literacy to understand study procedures (as assessed by self-report) or careless responding as indicated by the following:
- Failing ≥50% of the attention check items embedded in the screening questionnaires
- Overly rapid responding (ie, mean response time of <2 s for multiple-choice items)
- Qualitative review of long strings of identical entries
- Positive history of brain tumors, neurological disorders, or head injuries accompanied by the following:
- Loss of consciousness of >5 min
- >2 separate instances of clinically significant head trauma
- Recent alcohol or other substance use disorder (in the past 6 mo) or current psychosis (in the past 2 wk) according to the Psychiatric Diagnostic Screening Questionnaire [66]
- Past-month active suicidal ideation paired with either of the following:
  - An identified method, specific plan, or intent
  - Lifetime history of suicide attempts as assessed by the screen version of the Columbia Suicide Severity Rating Scale [67]

The exclusion criteria do not cover the presence of anxiety, depression, or other psychiatric symptoms, given our intention to target emotion dysregulation broadly and to assess the generalizability of effects across psychiatric diagnoses and severity, including participants with few or no symptoms and those without treatment history. However, the screening will include items from the self-rated screen version of the Columbia Suicide Severity Rating Scale (C-SSRS; for suicidal thoughts) as well as the alcohol, substance, and psychosis subscales from the Psychiatric Diagnostic Screening Questionnaire (PDSQ) [66]. Individuals who do not respond to the requisite screening items or do not meet eligibility requirements, including those whose responses to the psychiatric screening questions surpass established clinical thresholds, will be automatically informed that they will not be able to join the trial and will receive a list of mental health resources along with an invitation to contact our team should they desire additional referral assistance. We will provide further support for engaging with psychiatric care providers to respondents who endorse past-month active suicidal ideation with an identified method, specific plan, or intent on the C-SSRS.

#### Assessment

##### Overview

Each in-person assessment session will involve core measures expected to take 90 minutes, including a battery of computer-based behavioral tasks measuring cold and hot executive functions as well as the Positive and Negative Affect Schedule (PANAS) [68] to evaluate subjective mood before and after the neuropsychological examination. Within 24 hours of each session, participants will be asked to complete self-rated questionnaires to evaluate psychiatric symptoms and other variables of interest, which will require an additional 30 to 40 minutes. Table 1 presents key measures included at each assessment and intervention time point (refer to Multimedia Appendix 1 for a comprehensive list and additional details).

**Table 1:** Administration schedule of core measuresa.

Domains and measures			Format		Screening		Baseline assessment		Postwaitlist assessment	N-ACT^a^ assessment		Post–N-ACT assessment
**Emotion dysregulation**												
	Ruminative Responses Scale–Brooding	Self-report		✓				✓			✓	
	Three-Factor Impulsivity Index–Feelings Trigger Action	Self-report		✓				✓			✓	
	Three-Factor Impulsivity Index–Pervasive Influence of Feelings	Self-report				✓		✓			✓	
**Psychiatric symptom severity**												
	Columbia Suicide Severity Rating Scale	Self-report		✓								
	Demographic questionnaire or mental health history survey	Self-report		✓								
	Psychiatric Diagnostic Screening Questionnaire–Alcohol Use Disorder or Substance Use Disorder or Psychosis	Self-report		✓								
	Externalizing Spectrum Inventory–Revised	Self-report				✓		✓			✓	
	Inventory of Depression and Anxiety Symptoms, Expanded Version	Self-report				✓		✓			✓	
**Neuropsychological assessment battery**												
	Digit Span Backward	Behavioral				✓		✓			✓	
	Stop-signal task	Behavioral				✓		✓			✓	
	Trail-making test	Behavioral				✓		✓			✓	
	Wechsler Adult Intelligence Scale, Fourth Edition–Vocabulary	Interview				✓		✓			✓	
	Emotional stop-signal task	Behavioral				✓		✓			✓	
	Memory and affective flexibility task	Behavioral				✓		✓			✓	
**Affective control training (N-ACT)**												
	Adaptive emotional n*-*back task	Behavioral								✓		
	Emotional stop-signal task–adaptive	Behavioral								✓		

^a^A comprehensive list of secondary measures and additional outcomes is available in Multimedia Appendix 1.

##### Neuropsychological Battery

At each assessment session, participants will perform 5 computerized neuropsychological tasks (in a pseudorandom order) to index the hypothesized effects of N-ACT on emotional working memory and emotional response inhibition as well as the transfer of targeted inhibitory control processes from T1 (baseline time point) to T2 (postwaitlist or postintervention time point). The assessment battery accordingly includes 3 widely used gold standard cold executive functioning tasks that measure key components of cognitive control: (1) Digit Span Backward [69], a working memory test that requires participants to select (using the computer cursor) a series of numbers in the opposite order from which the digits were presented (in numerical strings of increasing length); (2) the stop-signal task (SST) [70], a measure of prepotent response inhibition in which participants must quickly indicate (via keypress) the direction of rapidly presented arrow stimuli, except on trials that include an unpredictable buzzer tone, which require termination of the initiated motor response; and (3) the trail-making test [71], an assay of set-shifting or task-switching ability that involves using the computer cursor to draw a path connecting an array of alphanumeric stimuli in an alternating sequential pattern (eg, “A” to “1” to “B” to “2,” and so on) as quickly as possible. The neuropsychological battery additionally includes the administration of the Vocabulary subtest from the Wechsler Adult Intelligence Scale, Fourth Edition [72], to assess crystallized intelligence, which we will examine in exploratory analyses of the specificity of intervention effects.

Participants will also complete 2 tasks to confirm the efficacy of N-ACT in augmenting 2 core facets of affective control, emotional response inhibition and emotional working memory: (1) the ESST [37,39,43], which follows procedures parallel to those of the traditional SST but differs in that participants must rapidly categorize affective pictures according to perceived valence (ie, “positive or pleasant” vs “negative or unpleasant”) instead of indicating the direction of arrow stimuli, while still inhibiting prepotent motor responses on a subset of trials with an unpredictable auditory stop signal (buzzer or tone); and (2) the memory and affective flexibility task (MAFT) [73], a novel modified n-back procedure that provides indices of emotional working memory as well as affective flexibility (as captured by emotional set shifting or task switching trials) by asking participants to recall whether they have previously seen (1-3 trials earlier) serially presented images with affective content (ie, “n-level,” which varies by block), with occasional interspersed “switch” trials prompting them to respond instead to perceived image valence (identical to ESST go or no-signal trials).

##### Psychophysiology

During the administration of the computerized cognitive assessment tasks, we will gather 2 metrics of arousal—pupil dilation, which reflects phasic norepinephrine signaling; and skin conductance, an index of autonomic sympathetic nervous system activity—to evaluate the physiological correlates of cold and hot executive functioning. We will consider incorporating physiological reactivity variables gathered during the latter set of tasks as secondary indicators in exploratory mechanistic analyses of the hypothesized treatment effects (Multimedia Appendix 1).

Pupil measurements will be recorded noninvasively with a Tobii T-120 infrared eye tracker [74] using E-Prime Extensions for Tobii [75]. We will not gather pupillometry data from participants who report a history of epilepsy or seizures triggered by arcade or video games, computer or television screens, or flickering fluorescent bulbs. Skin conductance levels will be monitored by electrodes placed (by the participant) on the inner sole of the foot. Trained research staff will calibrate physiological recording hardware (eg, by ensuring proper electrode placement and adjusting a chin rest to limit movement-related artifacts) for maximum comfort and signal.

##### EMA Surveys

We will use EMA to capture the temporal dynamics of real-world affect and regulation using a probes delivered via REDCap. Each EMA survey will ask participants to complete (1) PANAS items to rate current levels of anxiety, irritability, and sadness on a 7-point Likert scale; (2) the Momentary Ruminative Self-Focus Inventory–Abbreviated [76], a 3-item questionnaire evaluating state-level fluctuations in rumination, each rated on a 7-point Likert scale; and (3) the Momentary Impulsivity Scale [77], a 4-item questionnaire measuring momentary impulsivity on a 5-point Likert-type scale. Our chief EMA emotion dysregulation indices, which we predict will decline from the preintervention assessment to the postintervention assessment, include the (1a) average levels of, and (1b) variability (ie, mean square of successive difference) in, daily negative affect (derived from mean scores on the mood probes); (2) average daily levels of rumination via the Momentary Ruminative Self-Focus Inventory–Abbreviated; and (3) average daily levels of ERI, as measured by Momentary Impulsivity Scale scores during periods of elevated momentary negative affect (ie, scores above the person-level mean).

We will provide an in-person tutorial at baseline to help familiarize participants with the structure and content of EMA surveys, and to facilitate the completion of practice items. Participants will use their personal mobile phones or other internet-capable devices to submit EMA responses. Specifically, participants will receive automatic “push” notifications to complete EMA surveys 5 times per day. We will randomize notifications by binning participants’ available waking hours into 5 equal windows and then randomly selecting a survey time within each bin, with an interval of at least 90 minutes between notifications. Surveys will be available for completion for 80 minutes, and reminder prompts will be sent within 40 minutes of missed survey notifications.

##### Questionnaires

After each in-person assessment session, we will ask participants to complete questionnaires administered via REDCap (Table 1) as promptly as possible. Each set of questionnaires includes primary and secondary outcome measures related to emotion dysregulation as well as measures of psychiatric symptom severity, perceived functional impairment in cognitive abilities and activities of daily living. The baseline assessment will also include potential moderators of treatment effects for exploratory analyses. We will ask participants who request premature withdrawal from the trial to complete an identical assessment of the major outcomes of interest (ie, self-rated emotion dysregulation, psychiatric symptom severity, cognitive deficits, quality of life, and treatment acceptability) at the time of discontinuation.

#### N-ACT Intervention

##### Overview

The N-ACT program comprises 8 in-person training sessions over 2 months. The intervention is limited to weekly 1 h sessions to maximize adherence and minimize attrition. Each N-ACT session is guided by a trained “coach” (supervised by a licensed clinician) who will explain intervention procedures and rationale, offer relevant psychoeducation, and use motivational interviewing principles to encourage and support participants. At each training session, participants will practice 2 computer-based adaptive tasks targeting affective control. Approximately half of each N-ACT session will be spent performing these exercises, and the other half will be dedicated to coaching and assessment. Coaches will discuss standard cognitive behavioral psychoeducational content regarding emotion regulation (drawn from established therapy protocols).

##### Psychoeducational Content

To begin each weekly N-ACT session, a coach will review information from the previous session and discuss the implementation of learned techniques over the prior week. Coaches will then present information from 1 psychoeducational module, adapted from empirically supported interventions, on the topics presented in Textbox 2. Modules 2 to 7 will also include theoretical justification for cognitive training to enhance emotion regulation.

**Topics covered in coaching sessions.**

**Coaching session sequence**

1. Introduction and orientation, which includes an overview of session procedures and program structure, rationale, and personal goal setting
2. Cognitive behavioral approaches to emotion, which introduces fundamental concepts derived from cognitive behavioral therapy to elucidate links among affect, cognition, and behavior, plus an emotion regulation strategy (“STOP”) drawn from dialectical behavior therapy (DBT)
3. Environmental influences on feelings, in which participants identify risk and resilience factors that impede or facilitate emotional self-regulation, paired with distress tolerance skill adapted from DBT (“Self-soothing”)
4. Functional analysis of behavior, which involves functional analysis of problematic patterns of thought or behavior related to personal goals for emotion regulation
5. Mindfulness, involving a guided meditation practice
6. Psychological flexibility, which introduces key elements of acceptance and commitment therapy
7. Interpersonal skills, covering 2 closely related techniques (also borrowed from DBT) to promote adaptive emotional self-regulation and communication within relationships (“DEARMAN”)
8. Review, summary, and wrap-up, involving an examination of progress toward overarching treatment goals, review of key concepts, and solicitation of participant feedback on program components via interview and self-report

After engaging with psychoeducational content in each module, participants will complete the N-ACT tasks, and coaches will end each session with an informal debriefing to help consolidate learning and identify implementation intentions (ie, interim objectives toward participants’ identified treatment goal) for the coming week. Coaches will additionally administer the PANAS before and after the training tasks. We will audio record N-ACT training sessions (with participant consent) for regular review by independent raters to evaluate coach adherence and fidelity to the intervention guidelines using session-specific standardized forms created for this study.

##### N-ACT Task Battery

#### Overview

Participants will be asked to perform the same 2 computerized training tasks each week, in counterbalanced order: (1) an adaptive emotional n-back (AEnB) task targeting emotional working memory and (2) the ESST-adaptive (ESST-A), which targets emotional response inhibition. These are adaptive versions of the affective control behavioral measures completed during assessment sessions, modified to dynamically adjust the level of difficulty according to participant performance, which we expect to improve with additional training over time. Both training tasks include gamified graphical elements (eg, user-selected avatars) to facilitate user engagement (refer to Multimedia Appendix 1 for task instructions and other details).

#### The AEnB Task

The AEnB training task is an adaptive variant of procedures originally described by Levens and Gotlib [78,79], in which participants are instructed to indicate (as quickly and accurately as possible via keypress) whether a *target* affective picture stimulus is a “match” or a “mismatch” to a *cue* image shown *n* trials earlier, where *n* equals the number of interim trials between the presentation of the original cue and the to-be-evaluated target stimulus (ie, the “n-level”). The AEnB task additionally draws from an adaptive version of the dual-dimension emotional (n-back) working memory training task developed by Pan et al [53], such that cue-target pairs are only considered to match if the images are identical *and* spatially congruent, as each stimulus appears in 1 of 9 positions on an invisible 3×3 grid.

All AEnB task stimuli are pseudorandomly presented and counterbalanced to ensure equivalent numbers of matched and mismatched cue-target image pairs from 3 valence categories (neutral, negative, or positive) within each of 6 blocks, resulting in 36+n trials per block, or 216+n total trials. Image stimuli are shown for 1000 milliseconds on each trial, followed by a 2500-millisecond response window and a feedback screen shown for 1000 milliseconds. Trials are separated by a jittered intertrial interval of approximately 250 milliseconds (150-350 ms) assigned pseudorandomly on each trial.

The AEnB task involves 2 orthogonal adaptive components. The first is a tracking algorithm that adjusts the number of trials per condition (ie, emotional valence of cue-target pairs) to prioritize the encoding of spatial information (over affective content) into working memory for emotionally arousing images. Specifically, independent of the current n*-*level, higher accuracy for emotional (negative or positive) versus nonemotional (neutral) cue-target pairs will result in 2 fewer “match” trial pairs on the subsequent block. Crucially, the emotional images on these 2 trial pairs will remain identical but will be mismatched in spatial location. The algorithm will continue to swap matched emotional pairs with identical mismatched images until the participant achieves greater accuracy for neutral cue-target pairs (that are spatially congruent throughout the task), at which point the number of emotional trial pairs per condition returns to baseline, on a per-block basis, with 1 negative and 1 positive identical but spatially mismatched pair replaced by fully matched emotional cue-target pairs.

The second adaptive feature of the AEnB task is the n*-*level itself, which is adjusted after the first block either upward (n=3) or downward (n=2) at the start of each subsequent block (range: n=1-7), contingent on participants’ accuracy and response times (with larger values indicating greater working memory load). Specifically, the n-level decreases if a participant is unable to achieve at least approximately 80% accuracy (ie, at least 30 correctly identified pairs out of 36+n trials) within a given block; conversely, once a participant surpasses this accuracy threshold, the n-level increases at the start of the next block but only if the mean reaction time during trials with neutral target stimuli is faster than that during trials with emotional stimuli (regardless of whether cue-target pairs are matched or mismatched, provided that responses are accurate). This training approach is intended to encourage the development of relatively more rapid—but equally accurate—evaluative responses to less arousing or salient images. In sum, the AEnB task involves multiple adaptive components designed to facilitate diminished stimulus reactivity or enhanced engagement of affective control when processing emotionally evocative stimuli (eg, via enhanced attentional focus on key perceptual features rather than affective information, per se) for immediate recall in working memory.

#### The ESST-A Task

The ESST-A is a training variant of the original ESST [37,39,43], with comparable design elements; however, the ESST-A is distinct in that emotional (negative or positive) image stimuli are presented exclusively on no-go or stop trials (n=60 per valence), and, conversely, neutral images are only shown on go or no-signal trials (n=90 per block over 4 blocks) without an inhibitory cue. Participants must accordingly terminate emotional reactions to the most arousing and evocative stimuli on the ESST-A. This unique design feature of the ESST-A is meant to facilitate implicit associative learning, wherein salient affective content serves as a reliable “cue” that predicts upcoming stop signals, while stimuli with neutral information are consistently delivered without accompanying inhibitory demand. Therefore, repeated training with the ESST-A is expected to promote more automatic, reflexive engagement of inhibitory control at earlier stages of emotional information processing.

Similar to the nonadaptive version, the ESST-A comprises 4 blocks of 120 trials each, with stimuli presented pseudorandomly and counterbalanced across blocks to ensure equivalent numbers of trials per condition. Each ESST-A trial spans 2000 milliseconds, which includes a fixed pretrial pause period of 500 milliseconds, followed by a central fixation cross displayed for approximately 250 milliseconds, depending on a jittered latency value assigned on a trial-by-trial basis (150-350 ms), comparable to the AEnB task. The target image stimulus replaces the fixation cross in the center of the screen, where it is displayed for 1250 milliseconds. The initial (50 ms) stop signal is presented 250 milliseconds after stimulus onset, separately for positive and negative stimuli.

The primary adaptive component of the ESST-A is identical to the assessment version of the ESST (and similar to other SSTs). Specifically, the ESST-A includes 2 independent staircase algorithms that adjust valence-specific stop-signal delay (SSD) values (ie, distinct tracking values for negative and positive images) in 50-millisecond increments depending on participant performance, such that successful inhibition results in a longer SSD on the next no-go or stop trial with the same type of image, thereby increasing inhibitory demand. By contrast, commission errors (ie, false alarms) shorten each SSD by 50 milliseconds on subsequent no-go or stop trials with images from the same emotional valence category. These 2 stepwise algorithms are programmed to maintain commission error rates at approximately 50% of the no-go or stop trials, separately for negative and positive images, which enables calculation of valence-specific indices of emotional response inhibition (ie, stop-signal reaction time).

#### Data Quality Assurance

The screening survey and assessment questionnaires will randomly incorporate simple “attention checks” (eg, “2 + 2 = ?” and “for our data quality checking, please answer ‘1’ to this question”) to identify patterns of careless responding, which we will supplement by manual review to confirm the validity of self-report data. As previously noted, we will exclude participants who demonstrate patterns of careless responding during the screening questionnaire. Before conducting statistical analyses, research staff will perform comprehensive data cleaning and processing procedures as needed (eg, ascertaining violations of distributional assumptions for parametric tests and comparison with published norms).

#### Training and Reliability

Our team has comprehensive preparation procedures for all staff members involved in data collection. Before study recruitment, N-ACT coaches will receive extensive training in each session-specific intervention module to promote protocol adherence. Coach training and group supervision will comprise regular team-based meetings led by clinically licensed investigators, with didactic instruction, role-play, the development of interrater reliability to evaluate coach fidelity, and a review of practice tapes by senior researchers. We will conduct session adherence evaluations (as a group) monthly and provide retraining as necessary to address drift.

#### Risk Management

##### Overview

Trial procedures are designed to balance respect for participants’ autonomy with the need for safety. Study consent forms will provide contact information for crisis hotlines and other mental health resources. We will offer referral assistance to interested individuals. All team members with direct participant contact will receive training regarding psychiatric emergencies, suicide risk assessment, and crisis management. A detailed manual will provide assessment probes, decision rules, referral resources, and contact information for use during emergency situations. Training will emphasize procedures for providing feedback in a clinically sensitive manner, and senior investigators will use role-play to demonstrate fundamental principles related to assessment, obtaining supervision, providing referrals, and preparing documentation according to legal and ethical guidelines. This training will cover circumstances in which staff are permitted to breach confidentiality to contact emergency services.

Research assistants and coaches will be trained to carefully monitor cues indicating participant distress or discomfort, and we will offer breaks or opportunities to reschedule as warranted for fatigue or distress. Participants who endorse significant distress or endorse active suicidal ideation will be given the opportunity to speak with clinically licensed senior investigators, who will evaluate and determine the need for further follow-up. Research staff will work with these participants to develop a plan to obtain support, which may include strategies for discussing symptoms with their current provider, removing access to lethal means, and the provision of feedback or referrals. Our team has developed relationships with local sites to facilitate referral for individuals with insurance or those without; after referral, we will follow up with these participants as warranted to ensure that they received sufficient support.

##### Adverse Events

We will describe the limits of confidentiality as part of informed consent procedures to minimize the likelihood of study-related adverse events. During the consent process, we will also emphasize that our team is unable to provide emergency services or review research data in real time, and it is therefore important for participants to have access to other sources of care. Consent documents will encourage participants to contact the senior investigators with any concerns.

Throughout the trial, N-ACT coaches will also informally solicit information about potential issues at each training session. We will formally assess adverse events at the postintervention session. We will follow up with participants who show apparent clinical deterioration and will attempt to determine whether emergent symptoms or concerns might be somehow connected to study procedures. Adverse events will be reported to the university’s Committee for the Protection of Human Subjects within 1 week.

#### Measures

##### Screening

The screening survey will include a demographic questionnaire to assess inclusion criteria in addition to the self-report measures outlined in the following subsections.

###### Mental Health History Survey

The mental health history survey includes items to identify individuals whose linguistic abilities or cognitive, neurological, or psychiatric condition might interfere with completing study procedures or fully engaging in the intervention.

###### C-SSRS, Screen Version

The screening includes the self-report screen version of the C-SSRS [67], a well-validated and widely used instrument designed to assess suicidal thoughts and behaviors. Endorsement of the C-SSRS ideation item (Have you actually had any thoughts of killing yourself?) will trigger 3 follow-up items, to cover past-month presence of an identified method, intent, and a specific plan. Responding affirmatively to any of these 3 items will result in automatic study exclusion. Regardless of their responses, all participants will be asked an item to evaluate lifetime history of suicidal behavior; those who endorse this item in addition to past-month active suicidal ideation will also be automatically excluded from study participation.

###### PDSQ Prompts

The screening survey includes the Psychosis, Alcohol Use Disorder, and Substance Use Disorder scales of the PDSQ (6 *yes* or *no* items per scale) [66]. Questions regarding psychosis refer to the previous 2 weeks, and substance-related questions cover the previous 6 months. Prospective participants who respond affirmatively to at least 1 of these prompts (on any of the 3 PDSQ scales) will be automatically excluded, based on established clinical cutoffs for this measure [62].

###### Ruminative Responses Scale–Brooding

The Ruminative Responses Scale–Brooding (RRS-B) [13] will be used to assess study inclusion criteria. The RRS-B comprises 5 items to evaluate the dispositional propensity to engage in past-oriented patterns of repetitive negative thinking. Respondents rate prompts on a scale ranging from 1 (*almost never*) to 4 (*almost always*) based on characteristic reactions to dysphoric mood described by each item.

###### Three-Factor Impulsivity Index

Eligibility will be further determined using the Feelings Trigger Action (FTA) scale of the Three-Factor Impulsivity Index (TFII) [9], a 54-item multidimensional measure of trait-like impulsiveness that asks respondents to rate their agreement with each prompt on a scale of 1 (*I disagree a LOT*) to 5 (*I agree a LOT*). FTA includes 26 items describing tendencies to engage in impulsive speech and action in response to emotional states. The TFII includes 2 additional scales derived from factor analysis: Pervasive Influence of Feelings, which comprises 9 items reflecting powerful cognitive and motivational responses to emotion; and Lack of Follow Through, which captures distractibility and lack of perseverance. Pervasive Influence of Feelings is included as a secondary measure of emotion dysregulation (assessed in conjunction with FTA, at baseline).

##### Emotion Dysregulation

Our primary measures of emotion dysregulation will comprise self-reported rumination (RRS-B) and ERI (FTA). The secondary indices of emotion dysregulation further include EMA indices of daily negative affect, rumination, and ERI, in addition to scores on questionnaires capturing conceptually related constructs (Multimedia Appendix 1).

##### Psychiatric Symptom Severity

Participants will complete the following questionnaires at baseline, postwaitlist, and postintervention time points to evaluate the hypothesized clinical effects of N-ACT (refer to Multimedia Appendix 1 for the secondary symptom outcome measures).

##### Externalizing Spectrum Inventory–Revised

The Externalizing Spectrum Inventory–Revised (ESI-R) [80] is a 100-item questionnaire that covers 23 facets of externalizing psychopathology, including aggression, alcohol and other substance use problems, and fraudulent behavior. Respondents rate each ESI-R item as *true*, *mostly true*, *mostly false*, or *false*. Items referring to impulsivity (ie, redundant with the TFII) will not be included in the scale totals.

##### Inventory of Depression and Anxiety Symptoms, Expanded Version

The 99-item Inventory of Depression and Anxiety Symptoms, Expanded Version [81], is a validated questionnaire that assesses various emotional syndromes across the internalizing spectrum of psychopathology (eg, anxiety, depression, obsessions and compulsions). This instrument produces scores on 3 major factors (distress, fear, and mania), which are each composed of multiple subfactors (18 in total).

#### Statistical Analysis Plan

Our final target sample size (*n*=80) was derived from power analysis (1-β>0.85), based on hypothesized medium between-groups effect sizes (Cohen *d=*0.35) for the main outcomes of interest (α=.05). Before conducting the analyses, data will be checked for errors, statistical assumptions, and potential confounding variables to examine as covariates. Missing data imputation will also be performed as appropriate; we will present the results with and without imputed values for comparison. Our primary analyses will follow the International Conference on Harmonization E9 statistical principles for clinical trials. In accordance with these principles, we will use intention-to-treat analyses to estimate the main effects of N-ACT, using the final assessment scores of all randomized participants. To augment these findings, we will conduct sensitivity analyses restricted to data from participants who complete preintervention and postintervention assessments and at least 6 of the 8 weekly N-ACT sessions.

Descriptive statistics will be calculated to address the preliminary hypotheses regarding N-ACT’s acceptability and adherence. Mean scores on the Interest or Enjoyment and Value or Usefulness subscales of the adapted Intrinsic Motivation Inventory [82] will serve as our main indices of the N-ACT program’s acceptability (H1); specifically, we predict that participants who complete the intervention will provide average ratings of at least 5 (out of 7) across both scales. We further hypothesize that N-ACT participant adherence and retention will be comparable to those observed in other cognitive remediation interventions (H2); specifically, we anticipate median person-level adherence levels between 70% and 75%, which we expect to decrease at the group level by approximately 5% each week [63,64], with most participants (ie, >66%) completing all 8 N-ACT training sessions [33,52]. Consistent with other cognitive remediation protocol procedures [63], we will calculate percentage adherence for each participant as a function of the number of *completed* N-ACT training task trials divided by the *total* number of trials, that is, individual differences in the “dosage” of training.

We will consider potential confounds before testing key hypothesized effects. Of particular import, we will examine whether participants differ significantly in treatment gains based on assignment to a specific training coach and control for this unintended influence as necessary. Specifically, we will use multivariate analysis of covariance (MANCOVA) to explore the effect of coach assignment (independent variable) on postintervention emotion dysregulation (ie, scores on the TFII-FTA and RRS-B; dependent variables), controlling for scores at initial screening.

The core aim of this pilot trial is to determine whether N-ACT significantly reduces ERI and rumination, as measured by the TFII-FTA and RRS-B, respectively (H3). To achieve this aim and the related objective to explore potential mechanisms, we will conduct parallel 2 (group: immediate N-ACT vs waitlist control condition; between-subjects factor)×2 (time: T1 vs T2; within-subjects factor) mixed MANCOVAs to evaluate whether N-ACT completion is associated with greater pre-postintervention improvement in these 2 primary outcomes compared to the waitlist control condition. Among all participants, T1 will represent data collected at the baseline assessment; for participants assigned to the immediate N-ACT group, T2 refers to the postintervention assessment, whereas for those assigned to the control condition, T2 refers to the postwaitlist assessment. Thus, these models will not include postintervention data for participants initially allocated to the waitlist, permitting direct comparisons of the slope of change over time associated with completing N-ACT or the waitlist period. The key index of treatment effect in all MANCOVA models will be the group×time interaction. We will also perform a parallel set of multivariate cluster-robust general linear models with time (T1: baseline time point vs T2: postintervention time point) as the sole regressor on key outcomes of interest, which will include all participants who complete N-ACT (regardless of initial group or condition assignment) to maximize statistical power.

In addition to testing the hypothesized effects on emotion dysregulation, mixed MANCOVA and multivariate cluster-robust general linear models will be used to evaluate performance changes over time on (1) 2 hot executive functioning tasks (ESST and MAFT), which will be used to assess the hypothesized effects on the targeted aspects of affective control (H4); and (2) one hot (MAFT) and 2 cold (Digit Span Backward and SST) executive functioning tasks, which will be used to ascertain transfer to the nontargeted cognitive processes (H5). We will specifically derive key dependent variables from the ESST (valence-specific indices of stop-signal reaction time) and the MAFT (valence-specific indices of accuracy) to assess intervention-related changes in emotional response inhibition and emotional working memory, respectively. We will similarly calculate summary metrics of hot affective flexibility from the MAFT (valence-specific indices of accuracy and reaction time on less frequent “switch” trials prompting emotional reactions instead of working memory recall [73]) as well as cold working memory, updating from the Digit Span Backward (mean span) and cold response inhibition from the traditional SST (stop-signal reaction time) to evaluate near and far transfer of hypothesized N-ACT training effects, respectively.

Finally, we will perform a complementary set of secondary analyses (ie, mixed MANCOVA and multivariate cluster-robust generalized linear models) to explore the hypothesized downstream effects of N-ACT on changes in psychiatric symptom severity (H6). We will use dimension-reduction techniques (eg, confirmatory factor analysis) to extract the summary indices of internalizing and externalizing symptoms from the Inventory of Depression and Anxiety Symptoms, Expanded Version and ESI-R, respectively. Statistical models will use these latent factors as dependent variables, potentially providing preliminary evidence for the efficacy of affective control training as a transdiagnostic intervention to address psychopathology (via diminished emotion dysregulation; refer to Multimedia Appendix 1 for more information). Support for this hypothesis will motivate future work to more directly and precisely examine the effectiveness of the N-ACT program in sufficiently powered samples of patients with psychiatric conditions.

## Results

This trial is funded by support from the University of California Board of Regents and the Peder Sather Foundation (funding period: October 2022-September 2025). Recruitment is scheduled to begin in spring 2025. We will begin data analysis once data collection is complete, which is planned to occur in fall 2025. We anticipate publishing the findings in 2026.

## Discussion

### Summary

This randomized waitlist-controlled trial extends cognitive remediation research by testing a novel intervention program focused on facets of hot cognition typically unaddressed in this body of work. The trial will also be novel in testing a transdiagnostic sample comprising individuals with emotion regulation difficulties. Drawing on a substantial theoretical and empirical literature, we aim to modulate 2 aspects of affective control (ie, emotional working memory and emotional response inhibition) putatively underlying the problems with emotional self-regulation strongly implicated in common forms of psychopathology. Given the low cost, minimal coach training requirements, and relatively simple delivery of the N-ACT program, success in this project has major implications for reducing the public health burden associated with prevalent psychiatric conditions—as well as subclinical distress or dysfunction—involving tendencies toward rumination or ERI.

If our findings align with our hypotheses, this pilot trial will provide early data to support the further refinement and larger-scale deployment of N-ACT in a fully powered randomized clinical trial of patients with psychiatric conditions. Our findings will help identify the levels of interest and adherence and may suggest components that need to be refined for greater acceptability. Beyond the results concerning change in cognitive processes, we hope to learn more about how cognitive changes generalize to psychopathological outcomes. The profile of effects may help us understand which participants would be most likely to benefit from inclusion in future trials.

### Limitations

The potential impact of this work should be considered in the context of several limitations. Regarding the study sample, we will make every effort to maximize participant diversity and, accordingly, the generalizability of our findings; however, the modest target sample size (n=80) for this pilot trial will constrain our ability to ensure adequate representation of all psychiatric symptom types and severity levels. Relatedly, patients with acute symptoms of alcohol or substance use disorders and psychosis will be excluded. Additional studies will thus be necessary to elucidate the putative mechanisms identified in this work. Although our protocol incorporates a variety of well-validated multimodal measures to assess key constructs, 1 of the 2 primary behavioral outcomes will be derived from the MAFT, which is a novel task developed for this study. This limitation is somewhat lessened in that the MAFT includes design elements drawn from existing tasks that are widely used to index working memory and shifting and switching (eg, other emotional n-back procedures [73]). Finally, we see 2 core needs for enhancing the N-ACT intervention if the initial findings are promising. The current N-ACT intervention is limited in its scope of training around transfer to real-world tasks. Others have more extensively integrated ecological elements into cognitive remediation programs, including simulated work situations [83] and compensatory transfer sessions to supplement computerized training procedures [84]. Future work might help clients practice their skills in the context of mood inductions as a method to facilitate skills transfer. It will also be important to consider gamification [85,86] and other techniques to enhance the appeal of training programs.

### Conclusions

In conclusion, this study represents an important step in translating knowledge from basic affective science to produce interventions (with empirically informed treatment targets) that can be widely disseminated and easily implemented. This area of research is promising, given the need for alternative approaches to improve mental health because a substantial portion of patients do not seek nor respond to traditional treatment modalities. In addition, current rapid technological advancements, such as the proliferation of accessible and affordable mobile devices capable of delivering cognitive training, support the feasibility of digital therapeutics. Therefore, N-ACT has the potential to reduce barriers to mental health treatment, particularly among people from underserved groups or cultures with persistent stigma against talk-based psychotherapy as well as those who may be more amenable to computerized interventions for various reasons (eg, youth and patients with social phobia). Crucially, we expect N-ACT to have transdiagnostic applications, given that the program’s central aim is to address emotion regulation problems, which include shared features across a broad range of psychopathologies. We hope that establishing efficient and cost-effective means of modulating these common risk factors might eventually facilitate prevention among individuals considered vulnerable before diagnosable psychiatric disorders emerge.

## Appendix

Multimedia Appendix 1 Additional details on measures, tasks, and secondary analyses.

Multimedia Appendix 2 CONSORT-eHEALTH checklist (V 1.6.1).

## Abbreviations

AEnB: adaptive emotional n-back
C-SSRS: Columbia Suicide Severity Rating Scale
CONSORT-EHEALTH: Consolidated Standards of Reporting Trials of Electronic and Mobile Health Applications and Online Telehealth
EMA: ecological momentary assessment
ERI: emotion-related impulsivity
ESI-R: Externalizing Spectrum Inventory–Revised
ESST: emotional stop-signal task
ESST-A: emotional stop-signal task–adaptive
MAFT: memory and affective flexibility task
MANCOVA: multivariate analysis of covariance
MDD: major depressive disorder
N-ACT: neurobehavioral affective control training
PANAS: Positive and Negative Affect Schedule
PDSQ: Psychiatric Diagnostic Screening Questionnaire
REDCap: Research Electronic Data Capture
RRS-B: Ruminative Responses Scale–Brooding
SSD: stop-signal delay
SST: stop-signal task
TFII: Three-Factor Impulsivity Index

## Data Availability

The datasets generated and analyzed during this study will be available in this project’s Open Science Framework repository [87].
