# Supplementary material for: Cognitive Training for Emotion-Related Impulsivity and Rumination: Protocol for a Pilot Randomized Waitlist-Controlled Trial
Source: JMIR Res Protoc. 2025 Feb 19;14:e54221. doi: 10.2196/54221 (PMC11888066; doi:10.2196/54221)
Supplement: Multimedia Appendix 1 [file resprot_v14i1e54221_app1.docx]

## **Supplementary Materials**

Details concerning the full measure schedule, task instructions, and secondary analyses.

**Supplementary Table 1.** Full measure administration schedule.

|  | **Measure** | **Format** | **Screen**  **Assessment** | **Baseline**  **Assessment** | **Post**  **Waitlist**  **Assessment** | **N-ACT** | **Post-**  **N-ACT**  **Assessment** |  |
| --- | --- | --- | --- | --- | --- | --- | --- | --- |
| **Emotion dysregulation** | | | | | | | | |
|  | Ruminative Responses Scale-*Brooding* (RRS-B) | Self-report | ✓ |  | ✓ |  | ✓ |  |
|  | Three-Factor Impulsivity Index-*Feelings Trigger Action* (TFII-FTA) | Self-report | ✓ |  | ✓ |  | ✓ |  |
|  | Three-Factor Impulsivity Index-*Pervasive Influence of Feelings* (TFII-PIF) | Self-report |  | ✓ | ✓ |  | ✓ |  |
|  | Positive and Negative Affect Scales (PANAS)*^1^ | EMA/  Self-report |  | ✓ | ✓ | ✓ | ✓ |  |
|  | Momentary Impulsivity Scale (MIS)* | EMA |  | ✓ | ✓ |  | ✓ |  |
|  | Momentary Ruminative Self-focus Inventory-Abbreviated (MRSI-A)* | EMA |  | ✓ | ✓ |  | ✓ |  |
| **Psychiatric symptom severity** | | | | | | | | |
|  | Columbia Suicide Severity Rating Scale (C-SSRS) | Self-report | ✓ |  |  |  |  |  |
|  | Demographic questionnaire/  Mental Health History Survey (MHHS) | Self-report | ✓ |  |  |  |  |  |
|  | Psychiatric Diagnostic Screening Questionnaire-*Alcohol Use Disorder/*  *Substance Use Disorder/*  *Psychosis* (PDSQ) | Self-report | ✓ |  |  |  |  |  |
|  | Externalizing Spectrum Inventory-Revised (ESI-R) | Self-report |  | ✓ | ✓ |  | ✓ |  |
|  | Inventory of Depression and Anxiety Symptoms, Expanded Version (IDAS-II) | Self-report |  | ✓ | ✓ |  | ✓ |  |
|  | Adult ADHD Self-Report Scale (ASRS-v1.1)* | Self-report |  | ✓ | ✓ |  | ✓ |  |
|  | Eating Disorders Examination-Questionnaire (EDE-Q-13)* | Self-report |  | ✓ | ✓ |  | ✓ |  |
| **Neuropsychological assessment battery** | | | | | | | | |
|  | Digit Span Backwards (DSB) | Behavioral |  | ✓ | ✓ |  | ✓ |  |
|  | Stop-Signal Task (SST) | Behavioral |  | ✓ | ✓ |  | ✓ |  |
|  | Trail-Making Test (TMT) | Behavioral |  | ✓ | ✓ |  | ✓ |  |
|  | Wechsler Adult Intelligence Scales, Fourth edition -*Vocabulary* (WAIS-IV) | Interview |  | ✓ | ✓ |  | ✓ |  |
|  | Emotional Stop-Signal Task (ESST) | Behavioral |  | ✓ | ✓ |  | ✓ |  |
|  | Memory and Affective Flexibility Task (MAFT) | Behavioral |  | ✓ | ✓ |  | ✓ |  |
| **Affective control training (N-ACT)** | | | | | | | | |
|  | Adaptive Emotional *n-*Back  (AEnB) task | Behavioral |  |  |  | ✓ |  |  |
|  | ESST-Adaptive (ESST-A) | Behavioral |  |  |  | ✓ |  |  |
| **Additional measures & outcomes** | | | | | | | |  |
|  | Behavioral Inhibition/  Activation Scales (BIS/BAS)* | Self-report |  | ✓ |  |  |  |  |
|  | Risky Families Questionnaire (RFQ)* | Self-report |  | ✓ |  |  |  |  |
|  | Sound Sensitivity Symptoms Questionnaire (SSSQ)* | Self-report |  | ✓ |  |  |  |  |
|  | Adapted Intrinsic Motivation Inventory (AIMI)^2^ | Self-report |  |  |  | ✓ |  |  |
|  | “Wheel of Fortune” task* | Behavioral |  |  |  | ✓ |  |  |
|  | Pupillometry* | Physiology |  | ✓ | ✓ |  | ✓ |  |
|  | Skin conductance level* | Physiology |  | ✓ | ✓ |  | ✓ |  |

*Indicates secondary measures and outcomes for exploratory mechanistic analyses.

^1^The full PANAS will be administered twice at each in-person session; participants will also receive momentary negative affect probes adapted from the PANAS during ecological momentary assessment (EMA).

^2^The AIMI will be administered at the conclusion of the eighth/final N-ACT session.

### Secondary Measures & Outcomes

***Adapted Intrinsic Motivation Inventory (AIMI; [1]).*** *The AIMI is a questionnaire evaluating participants’ subjective impressions of study procedures, which we have modified for this trial to assess overall satisfaction with the intervention, the training coach, N-ACT tasks, and skills utilization. Participants will be asked to complete the AIMI at the final N-ACT session along with an informal debriefing interview performed by a research team member.*

***Positive and Negative Affect Scales (PANAS;* [2]*).*** The PANAS is a 22-item measure of self-reported positive affect (e.g., *enthusiastic*, *proud*) and negative affect states (e.g., *nervous*, *upset*). Each item is rated according to the degree respondents are presently experiencing each emotion, on a 1 (“*very slightly or not at all*”) to 5 (“*extremely*”) Likert-type scale. We will ask participants to complete PANAS mood ratings twice during each in-person (assessment and intervention) session to capture mood states before and after behavioral task procedures. Exploratory analyses will use the PANAS to examine affective shifts surrounding neuropsychological assessment and cognitive training.

#### Psychiatric Symptom Severity

***Adult ADHD Self-Report Scale (ASRS-v1.1;* [3]*).*** The ASRS-v1.1 is a brief symptom checklist that evaluates recent history of attention-deficit/hyperactivity disorder (ADHD) symptoms in adults. Respondents answer each of 18 items, which correspond to DSM ADHD diagnostic criteria, according to frequency (“*never*” to “*very often*”) over the preceding six months. Although there are no established clinical cutoffs and the ASRS-v1.1 does not generate scale scores, it has acceptable psychometric properties and the first six items are especially valid predictors of ADHD diagnoses in non-clinical samples.

***Eating Disorders Examination-Questionnaire-13 (EDE-Q-13;* [4,5]*).*** The EDE-Q-13 is a 13-item self-rated assessment of feeding and eating pathology. It is a shortened version of the original EDE-Q measure [S3], which was itself derived from a “gold-standard” clinical interview to determine the presence and severity of eating disorder symptoms. Respondents receive scores on the following scales/subscales: *Global*; *Restraint*; *Eating Concerns*; *Shape Concerns*; and *Weight Concerns*.

#### Potential Moderators

***Behavioral Inhibition/Activation Scales (BIS/BAS;* [6]*).*** The BIS/BAS is a 24-item questionnaire that we will administer as part of the baseline online assessment. This self-report instrument was developed to measure individual differences in sensitivity to reward and punishment. Respondents are asked to rate each item on a four-point scale (from “*very true for me*” to “*very false for me”*), which produces scores on a general *Behavioral Inhibition* scale as well as three *Behavioral Activation* scales: *Drive*, *Fun-seeking*, and *Reward Responsiveness*. We aim to explore how approach and avoidance tendencies (as measured by the BIS/BAS) relate to behavioral task performance and intervention response.

***Risky Families Questionnaire (RFQ;* [7]*).*** History of early life adversity will be measured using the RFQ, which we will administer online as part of the baseline questionnaires. The RFQ is a 10-item measure (with three “filler” items) designed to assess the degree of physical, mental, and emotional stress that subjects faced in their homes from ages 5-15. Each item is rated on a five-point Likert scale based on the extent to which they agree with items pertaining to their family functioning during this time period. Given the well-established role of adverse childhood experiences in psychopathology risk, we intend to explore whether such exposure influences predicted intervention effects.

***Sound Sensitivity Symptoms Questionnaire (SSSQ;* [8]*).*** This five-item survey requires respondents to indicate how frequently they have experienced symptoms of auditory hyperacusis (e.g., misophonia) in the preceding two weeks. Response options range from “*0-1 days*” to “*11-14 days*”, corresponding to item scores of 0-3, which are summed to yield a total score indicating overall level of distress and functional impairment caused by intolerance of certain everyday sounds. This brief inventory has strong psychometric properties in treatment-seeking adults [S6]. SSSQ data will be used for exploratory purposes to examine the specificity of intervention effects to psychiatric syndromes versus related conditions involving inhibitory deficits.

***Reinforcement learning assessment.*** Following the training portion of each N-ACT session, we will ask participants to complete a gamified assessment task that is implemented in an available smartphone application. The “Wheel of Fortune” game is a brief (< 5 minutes to complete) task for probing reinforcement learning and the impact of momentary mood on decision-making. Participants learn, through trial and error, the rate at which two stimuli yield reward (points). There are two learning blocks, one before and one after a mood-inducing event (a “wheel of fortune”, in which they win or lose a much larger number of points). Participants ultimately choose between options they may have learned about under different affective states in order to maximize accrual of points, which allows us to quantify the extent that changes in mood impact reward perception in each session, and to evaluate whether this propensity is impacted by the intervention (in exploratory analyses).

### N-ACT task instructions

##### The AEnB task

### **“**Welcome to the Mind's Eye Detective Agency, where your keen observation and memory skills help solve mysteries that baffle others. As our newest recruit, you'll undergo specialized training exercises to sharpen your abilities to notice what others overlook, especially in emotionally charged situations.

### In each case, you'll be presented with image scenes from various ongoing investigations. Your job is to identify whether the scene before you is an exact 'MATCH' or 'MISMATCH' to a previous scene you encountered in an earlier case.

### (1) Press the 'Z' key (marked with a green sticker) if the image is an identical MATCH.

### (2) Press the 'M' key (marked with a red sticker) if the image is a non-identical MISMATCH.

You should compare the scene for each case to the one that was shown between 1 and 7 cases earlier. Use the ‘*n*-level’ displayed in the top right corner of your screen to determine how many cases earlier in the sequence you should remember the scene for comparison.

For example, the n-level for the first case roundup is two (*n* = 2), so you should press the ‘match’ key ('z') ONLY when shown a scene that is EXACTLY THE SAME as the scene you saw two cases earlier. If the scenes are not totally identical, you should press the ‘mismatch’ key ('m').

IMPORTANT: Pay attention to every detail, including location, as even the smallest clue can crack a case wide open. Remember, accuracy and speed are your best tools in solving these mysteries.

Press <SPACEBAR> when you are ready to solve your first case.”

##### The ESST-A

“WELCOME TO EQUILIBRIUM: The Realm of Emotional Balance

Before we begin, choose your avatar. Each represents a unique strength and emotional energy…

…In the mystical world of Equilibrium, the balance of emotions governs the harmony of life. As a chosen traveler, you will embark on a quest to master the essence of emotions, using your instinct and swift judgment to navigate through the emotional landscapes that lie before you. Your mission is vital – for your actions will either restore or disrupt the delicate balance of this world.

Your Quest: You are about to undergo a series of Perception Training Missions, designed to refine your ability to discern the emotional essence of Equilibrium's myriad elements. Each encounter will present you with an image, an echo of emotions waiting for your judgment.

In each Perception Training Mission, your goal is to quickly and accurately determine the emotional essence of the pictures you encounter. Upon the appearance of each image, you must quickly decide its emotional nature:

POSITIVE (pleasant) or NEGATIVE (unpleasant).

- Press the 'A' key (marked with a smiling sticker) for images that radiate positivity.

- Press the 'L' key (marked with a frowning sticker) for images that exude negativity.

If an image's emotional essence is not immediately clear, trust your instinct or 'gut reaction'. Your swift and accurate responses are crucial for maintaining the balance in Equilibrium.

As you journey through Equilibrium, you will be tested by Mastery Challenges. These are moments when the realm itself will challenge your emotional control by emitting a distinct sound – the STOP-SIGNAL. When this signal resonates, you must halt your action – a true test of your mastery over impulse and emotion.

Key Points for Your Journey:

- Do not await the STOP-SIGNAL. Reacting to each image you encounter with both speed and accuracy is paramount. If you delay, anticipating the STOP-SIGNAL, the challenges ahead will intensify, demanding even greater control.

- Should you hear a STOP-SIGNAL, either as the image appears or thereafter, you must cease your judgment. This restraint, regardless of the timing, is essential for your success.

- Remember: Press 'A' for POSITIVE or PLEASANT scenes and press 'L' for NEGATIVE or UNPLEASANT scenes you encounter during each Perception Training Mission.

It is time to prepare for the journey ahead. Your actions and decisions carry the weight of balance; embrace this quest with both speed and precision.

Press <SPACEBAR> to embark on your first Perception Training Mission and to begin your journey towards mastering the emotional balance of EQUILIBRIUM.”

### Exploratory Analyses

Although we will be underpowered, we plan to test a mediational model (see Supplementary Figure 1) at a descriptive level to facilitate planning future studies. Exploratory mediation analyses will use structural equation modeling (SEM) to examine whether intervention-related improvements in affective control are linked to accompanying decreases in psychiatric symptom severity via diminished emotion dysregulation. In a preliminary step, we will perform confirmatory factor analyses to test our hypothesized measurement model of three latent variables measured at baseline/postwaitlist: *Affective Control, Emotion Dysregulation,*and psychopathology severity*.*Our multimodal assessment approach is intended to produce several indicators for each latent factor; however, we will evaluate separate models for these observed indicators if they fail to converge as expected, and explore alternative measurement models that incorporate baseline/postwaitlist scores from (a) psychophysiology metrics and (b) conceptually-relevant secondary measures highlighted in the previous (“Measures”) section. We will identify the best-fitting structural model using preintervention (baseline/postwaitlist) scores to constrain factor loadings in a parallel set of postintervention indicators for mediation analyses. In sum, we plan to preliminarily test a hypothesized mediation model that will use postintervention *Affective Control* as a predictor of postintervention outcomes and postintervention *Emotion Dysregulation*as a mediator, while including preintervention scores for each construct as autoregressive predictors and allowing for timepoint-specific covariance among the three latent factors.

## **Supplementary References**

1. Ryan RM. Control and information in the intrapersonal sphere: an extension of cognitive evaluation theory. J Pers Soc Psychol 1982;43(3):450-461. [doi: 10.1037/0022-3514.43.3.450]
2. Watson D, Clark LA, Tellegen A. Development and validation of brief measures of positive and negative affect: the PANAS scales. J Pers Soc Psychol 1988;54(6):1063. [doi: 10.1037//0022-3514.54.6.1063]
3. Kessler RC, Adler LA, Ames M, et al. The World Health Organization Adult ADHD Self-Report Scale (ASRS): a short screening scale for use in the general population. Psychol Med 2005 Feb;35(2):245-256. [doi: 10.1017/s0033291704002892]
4. Lev-Ari L, Bachner-Melman R, Zohar AH. Eating Disorder Examination Questionnaire (EDE-Q-13): Expanding on the short form. J Eat Disord 2021 Apr 29;9(1):57. [doi: 10.1186/s40337-021-00403-x]
5. Fairburn CG, Beglin SJ. Assessment of eating disorders: Interview or self‐report questionnaire? Int J Eat Disord 1994 Dec;16(4):363-70. [PMID: 7866415]
6. Carver CS, White TL. Behavioral inhibition, behavioral activation, and affective responses to impending reward and punishment: the BIS/BAS scales. J Pers Soc Psychol 1994 Aug;67(2):319-333. [doi: 10.1037/0022-3514.67.2.319]
7. Taylor SE, Lerner JS, Sage RM, Lehman BJ, Seeman TE. Early environment, emotions, responses to stress, and health. J Pers 2004 Dec;72(6):1365-1393. [doi: 10.1111/j.1467-6494.2004.00300.x]
8. Aazh H, Hayes C, Moore BCJ, Danesh AA, Vitoratou S. Psychometric evaluation of the Hyperacusis Impact Questionnaire (HIQ) and Sound Sensitivity Symptoms Questionnaire (SSSQ) using a clinical population of adult patients with tinnitus alone or combined with hyperacusis. J Am Acad Audiol 2022;33:248-258. [doi: 10.1055/a-1780-4002]
